# Mitogen-activated protein kinase binding protein 1 (*MAPKBP1*) is an unfavorable prognostic biomarker in cytogenetically normal acute myeloid leukemia

**DOI:** 10.18632/oncotarget.3519

**Published:** 2015-03-10

**Authors:** Lin Fu, Jinlong Shi, Kai Hu, Jijun Wang, Weidong Wang, Xiaoyan Ke

**Affiliations:** ^1^ Department of Hematology and Lymphoma Research Center, Peking University, Third Hospital, Beijing, China; ^2^ Medical Engineering Support Center, Chinese PLA General Hospital, Beijing, China

**Keywords:** MAPKBP1, prognostic biomarker, CN-AML

## Abstract

Mitogen-activated protein kinase binding protein 1 (*MAPKBP1*) is a key transcription factor in the *NF-κB* signalling pathway. In this study, associations between *MAPKBP1* expression and molecular and clinical characteristics were evaluated by several microarray datasets. We found that *MAPKBP1* was over-expressed in cytogenetically normal AML (CN-AML) patients compared to normal bone marrow. High *MAPKBP1* expression (*MAPKBP1*^high^) was associated with significantly shorter event-free survival (EFS; *P* = 0.0004) and overall survival (OS; *P* = 0.0006) than low *MAPKBP1* expression (*MAPKBP1*^low^) in a cohort of 157 CN-AML patients. In multivariable analyses, *MAPKBP1*^high^ remained associated with shorter EFS (*P* = 0.003) and OS (*P* = 0.01). Validation in an independent cohort of 162 CN-AML patients further confirmed the prognostic value of *MAPKBP1* (OS, *P* = 0.00172). Gene-expression profiling revealed that some important oncogenes, including *MYCN*, *MYB, CDK6* and *CCND2*, etc, were up-regulated, while cell signalling pathways leading to apoptosis, antigen processing, and natural killer cell-mediated cytotoxicity were down-regulated in *MAPKBP1*^high^ patients with CN-AML. MicroRNA expression profiling revealed thatsome oncogenic microRNAsincluding *miR-155* and *miR-126* were up-regulated, whilst anti-oncogenic microRNAsincluding *miR-148a* and *miR-193a* were down-regulated in *MAPKBP1*^high^ patients with CN-AML, which may underlie the pathological processes in this malignancy. Taken together, these findings suggest *MAPKBP1*^high^is a novel, unfavourably prognostic biomarker for CN-AML risk-stratification.

## INTRODUCTION

Cytogenetically normal AML (CN-AML) is the most commonly encountered primary AML, yet their clinical prognosis are sharply heterogeneous and it lacks effective prognostic indicators [1]. Although the leukemic blasts of CN-AML patients do not contain detectable chromosome abnormalities by microscope, they still harbour mutations and aberrantly expressed genes, microRNAs and changes in DNA methylation that are potential prognostic markers [2-4]. For example, mutations in *NPM1* [5] and *CEBPA* [6] are associated with favourable outcomes; whereas mutations in *FLT3-ITD*[7], *WT1* [8], *ASXL1* [9], *MLL* [10], *RUNX1* [11], *TET2* [12] and *DNMT3A* [13] are associated with an unfavourable prognosis. High expression levels of *WT1* [14], *BAALC* [15], *ERG* [16], *MN1* [17], *DNMT3B* [18], and *TCF4* [19] as well as low expression of *LEF1* [20] have also been shown to be unfavourable prognostic factors, as has the high expression of *miR-155* [21] and *miR-3151* [15], and low expression of *miR-181a* [22, 23].

The *NF-κB* signalling pathway plays an important role in solid tumors and hematologic malignancies, including CN-AML [24-26]. Recent findings suggested that *MAPKBP1* acted as a scaffold protein interacting with TNF-receptor associated factor 2 (*TRAF2*) and TGF-β-activated kinase1 (*TAK1*). *MAPKBP1* could facilitate the polyubiquitination of *TRAF2*, leading to the TAK1 mediated activation of *NF-κB* [27, 28]. According to the role of *NF-κB* in the pathogenesis of CN-AML, it was speculated that the expression of *MAPKBP1* might be related to prognosis in patients with CN-AML.

We found not only *MAPKBP1* was highly expressed in CN-AML compared to normal bone marrow (BM) when measured using microarray, but also *MAPKBP1*^high^ was an unfavourably prognostic factor in patients with CN-AML amongst 2 independent, large AML patient cohorts. In addition, the first evidence showed that expression of *MAPKBP1* was associated with distinct molecular and clinical characteristics. In order to further elucidate its function, we also identified *MAPKBP1* associated genes in the genome wide scale, as well as changes in microRNA expression and DNA methylation profiles.

## RESULTS

### Expression of *MAPKBP1* in CN-AML cells and normal BM

**Figure 1:**
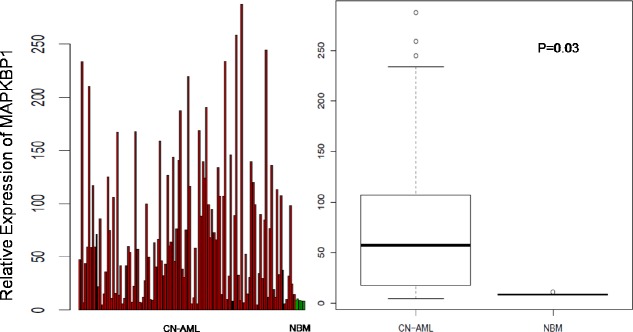
Expression of *MAPKBP1* in CN-AML patients and normal bone marrow Relative expression of *MAPKBP1* in 116 CN-AML cases compared with 5 normal bone marrow samples.

We analysed *MAPKBP1* expression in CN-AML and normal BM using a microarray assay. Both CN-AML (n = 116) and normal BM (n = 5) expressed *MAPKBP1*, although there was a relatively higher expression of *MAPKBP1* in the former (*P* = 0.03) (GEO accession number *GSE1159*) [29]. These findings indicated that *MAPKBP1* was widely expressed at a high level in CN-AML, and easy to detect. (Figure 1A and 1B).

### Association of *MAPKBP1* expression levels with pre-treatment patient characteristics

In the cohort of 157 CN-AML patients, patients with M1 disease were more likely to have *MAPKBP1*^high^ in the FAB subtype (*P* = 0.05). *MAPKBP1*^high^ patients were more likely to carry a *FLT-ITD* mutation (*P* < 0.001) than *MAPKBP1*^low^ patients. We found no association between *MAPKBP1* expression and other gene mutations, but *MAPKBP1*^high^ patients with CN-AML were more likely to have a high expression of *ERG1*, *WT1*, *DNMT3B* and *TCF4* (*P* < 0.001, *P* < 0.001, *P* < 0.001, and *P* < 0.001, respectively). In addition, there was also a significant difference between the occurrence of the ELN genetic favourable group in the *MAPKBP1*^high^ and *MAPKBP1*^low^ groups (*P* = 0.001). (Table 1).

**Table 1:** Patients' characteristics in the CN-AML cohort according to the *MAPKBP1* expression

Variable	*MAPKBP1*^high^, n=78	*MAPKBP1*^low^, n=79	P
Median age. y (range)	48.50 (18-77)	51 (16-73)	0.256
Female sex, no.(%)	37 (47.4)	36 (45.6)	0.87
FAB subtype, no.			
M0	1	2	1
M1	28	17	0.05
M2	19	13	0.24
M3	1	0	0.50
M4	13	11	0.66
M5	14	25	0.06
M6	0	1	1
Other	2	10	0.03
FLT3-ITD, no.	50	16	<0.001
FLT3-TKD, no.	10	10	1
NPM1, no.	46	36	0.11
CEBPA, mutated, no.			
Single	4	4	1
Double	5	11	0.18
N-RAS, mutated, no.	4	9	0.25
K-RAS, mutated, no.	0	1	1
IDH1, mutated, no.	10	9	0.81
IDH2, mutated, no.	5	8	0.81
ELN genetic group, no			
Favorable	19	40	0.001
Intermediate-I	68	54	0.19
High ERG, no.	51	27	<0.001
High BAALC, no.	44	34	0.11
High LEF1, no.	33	45	0.35
High MN1, no.	42	36	0.34
High WT1, no.	54	24	<0.001
High DNMT3B, no.	55	23	<0.001
High TCF4, no.	54	24	<0.001

CN-AML indicates cytogenetically normal acute myeloid leukemia; FAB, French-American-British classification; ITD, internal tandem duplication; ELN, European Leukemia Net; and TKD, tyrosine kinase domain.

High *ERG, BAALC, LEF1, MN1, WT1, DNMT3B* and *TCF4* expression were defined as an expression level above the median of all samples, respectively.

### *MAPKBP1*^high^ is associated with unfavourable treatment

**Figure 2:**
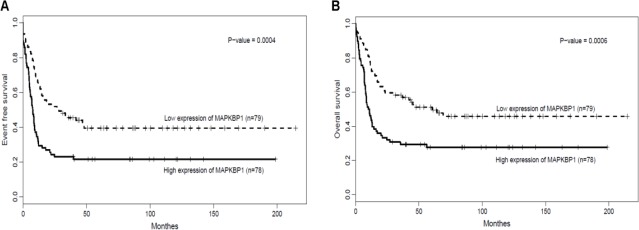
*MAPKBP1*^high^ is associated with unfavourable treatment (A) EFS and (B) OS in the entire cohort of 157 CN-AML cases.

As a whole, the median OS and EFS for *MAPKBP1*^high^ group were significantly shorter than that of *MAPKBP1*^low^ patients. (*P* = 0.007, *P* = 0.004, respectively. See Table 2). While for the comparison of Log-rank test in different divisions according to *MAPKBP1* expression, *MAPKBP1*^high^ group also had significantly shorter EFS (Figure 2A, *P* = 0.0004) and OS (Figure 2B, *P* = 0.0006) compared to the *MAPKBP1*^low^ group. (Table 2).

**Table 2:** Survival according to *MAPKBP1* expression in all patients and European Leukemia Net Genetic Groups

Outcome	All patients, n=157			ELN Favorable group			Intermediate-I		
	MAPKBP1^high^, n=78	MAPKBP1^low^, n=79	P	MAPKBP1^high^, n=19	MAPKBP1^low^, n=40	P	MAPKBP1^high^, n=68	MAPKBP1^low^, n=54	P
OS									
Median OS, m	10.46 (0.07-198.7)	43.47 (0.13-214.5)	0.00 7	20.01 (1.05-163.10)	52.28 (0.3-214.5)	0.2 7	8.49 (0.07-198.7)	39.54 (0.13-190.3)	0.04
Estimated OS at 3 y. % (95% CI)	0.29 (0.21-0.42)	0.58 (0.48-0.70)	0.01	0.42 (0.25-0.71)	0.63 (0.49-0.80)	0.1 9	0.264 (0.18-0.4)	0.56 (0.44-0.71)	0.04
EFS									
Median EFS, m	7.64 (0.03-198.7)	28.12 (0.03-214.5)	0.00 4	11.93 (0.03-131.9)	40.48 (0.03-214.5)	0.2 1	6.83 (0.03-198.7)	24.94 (0.03-190.3)	0.00 9
Estimated EFS at 3 y. % (95% CI)	0.23 (0.15-0.35)	0.46 (0.36-0.58)	0.00 2	0.37 (0.20-0.66)	0.55 (0.42-0.73)	0.0 6	0.19 (0.12-0.31)	0.41 (0.29-0.56)	0.00 3

OS, overall survival; CI, confidence interval; EFS, event-free survival.

### Associations of *MAPKBP1* expression with clinical outcome in ELN genetic groups

**Figure 3:**
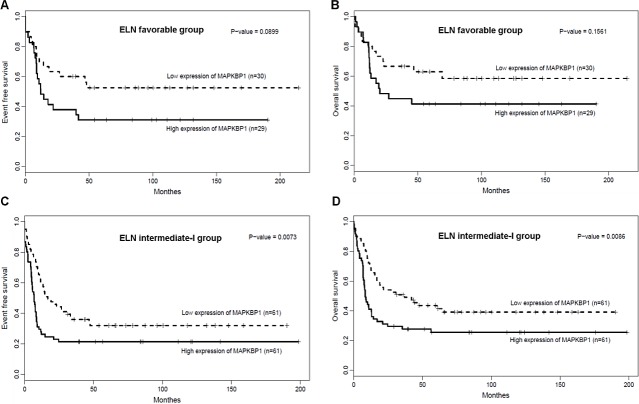
Associations of *MAPKBP1* expression with clinical outcome in ELN genetic groups (A) EFS and (B) OS of CN-AML patients in the ELN favourably genetic group. (C) EFS and (D) OS of CN-AML patients in the ELN intermediate-I genetic group.

We analysed the associations between *MAPKBP1* expression and outcome separately within the ELN favourable and Intermediate-I genetic groups. Within the ELN favourable group (n = 59), there was no significant difference in EFS (Figure 3A, *P* = 0.0899) and OS (Figure 3B, *P* = 0.1561) between *MAPKBP1*^high^ group and *MAPKBP1*^low^group. However, *MAPKBP1*^high^ group tended to have shorter EFS and OS than *MAPKBP1*^low^ group. In the ELN Intermediate-I group (n = 122), *MAPKBP1*^high^ group had a shorter EFS (Figure 3C, *P* = 0.0073) and shorter OS (Figure 3D, *P* = 0.0086) than *MAPKBP1*^low^ group. Median OS and EFS of different expressing divisions also showed a significant difference. (Table 2).

### *MAPKBP1* expression is associated with shorter EFS and OS in multivariable analyses

After adjusting for the impact of several known risk factors, we performed multivariable analyses to determine the prognostic significance of *MAPKBP1* expression. In the multivariable model of EFS, *MAPKBP1*^high^ group had a shorter EFS (*P* = 0.009, Table 3). The other factors associated with shorter EFS were the *NPM1* wild type and *FLT3-ITD* genotypes. In a multivariable model for OS, *MAPKBP1*^high^ group had a shorter OS (*P* = 0.01, Table 3). The other factors associated with shorter OS were the *NPM1* wild type and *FLT3-ITD* genotypes.

**Table 3:** Multivariable analysis with EFS and OS for the CN-AML patients

Variable	OS, n=157		EFS, n=157	
	HR (95% CI)	P	HR (95% CI)	P
MAPKBP1 expression, high vs low	1.87 (1.20-2.91)	0.006	1.87 (1.23-2.84)	0.003
Age, per 10-y increase	1.17 (1.00-1.35)	0.036	1.08 (0.95-1.24)	0.251
Sex, male vs female	0.82 (0.54-1.24)	0.35	0.99 (0.67-1.46)	0.962
NPM1, mutated vs wild type	0.5 (0.31-0.79)	0.003	0.52 (0.34-0.81)	0.004
FLT3-ITD, mutated vs wild type	1.77 (1.10-2.85)	0.018	1.63 (1.04-2.55)	0.033
CEBPA, mutated vs wild type	0.64 (0.34-1.22)	0.174	0.71 (0.39-1.28)	0.258

CN-AML indicates cytogenetically normal acute myeloid leukemia; RFS, relapse-free survival; OS, overall survival; EFS, event-free survival; HR, hazard ratio; CI, confidence interval; and ITD, internal tandem duplication;

### Validation in a large and independent cohort of CN-AML samples

We studied an independent cohort of 162 previously untreated CN-AML patients. In the validating cohort, patients with M1 and M6 disease were more likely to have *MAPKBP1*^high^ in the FAB subtype (*P* = 0.001, *P* = 0.00284, respectively).We also found that *MAPKBP1*^high^ patients with CN-AML were more likely to have a higher expression of *ERG1*, *MN1*, *WT1*, *DNMT3B* and *TCF4* (*P* < 0.001, *P* = 0.028, *P* < 0.001, *P* < 0.001, and *P* < 0.001, respectively) and low LEF1 (*P* < 0.001) compared with *MAPKBP1*^low^ patients (supplemental Table S1). In addition, *MAPKBP1*^high^ patients showed a significantly shorter OS (n=81 vs n=81, *P* = 0.00172; supplemental Figure 1) than *MAPKBP1*^low^ patients in the validating cohort.

### Genome-wide gene-expression profiles associated with *MAPKBP1* expression

**Figure 4:**
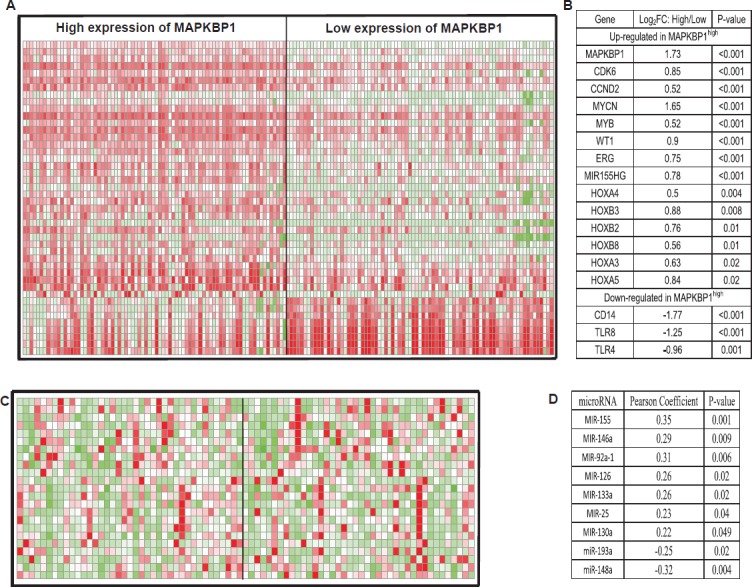
Genes and microRNAs associated with *MAPKBP1* expression (A) expression heatmap of associated genes (B) the list of associated genes. (C) expression heatmap of associated microRNAs (D) the list of associated microRNAs.

In order to further evaluate the role of *MAPKBP1* in CN-AML, we derived *MAPKBP1*-associated gene-expression profiles using a microarray analysis. We identified 571 up-regulated genes and 757 down-regulated genes that were significantly associated with *MAPKBP1*^high^ (supplemental Table S2). The up-regulated genes included some of those previously found to be involved in AML, including *CDK6* and *CCND2* that encode a cyclin kinase, *MYCN*, *MYB*, *WT1*, members of the *HOX* gene family (*HOXB2, HOXB3, HOXB8, HOXA3, HOXA4*, and *HOXA5*) that encode transcription factor proteins, and c-kit that encodes a tyrosine kinase. *ERG*, an independent unfavourable prognostic factor in CN-AML, was also up-regulated. *MiR-155* host gene up-regulation in *MAPKBP1*^high^ CN-AML was unexpected as this microRNA was previously found to function as an oncogene in CN-AML [21]. The down-regulated genes included those involved with both normal differentiation gene of monocyte/macrophage including *CEBPB* and immune function including *CD14*, *TLR4*, and *TLR8* (Figure 4). These provided further support for the correlation described above.

The *MAPKBP1*-associated cell signalling pathways were evaluated by MSigDB [30] in order to assess the biological features of the expression profile of *MAPKBP1* (Table 4). Signalling pathways involved in apoptosis, antigen processing and natural killer cells mediated cytotoxicity were down-regulated (*P* = 0.024, *P* < 0.001, and *P* = 0.007, respectively). These findings were consistent with the above noted dysregulated genes involved in the development of CN-AML.

**Table 4:** Cell signalling pathways associated with *MAPKBP1* expression levels

Pathway name	According to high expression of *MAPKBP1*	
	Regulation	P
KEGG_CHEMOKINE_SIGNALING_PATHWAY	Down	0.044
KEGG_UBIQUITIN_MEDIATED_PROTEOLYSIS	Up	0.021
KEGG_APOPTOSIS	Down	0.024
KEGG_ANTIGEN_PROCESSING_AND_PRESENTATION	Down	<0.001
KEGG_NATURAL_KILLER_CELL_MEDIATED_CYTOTOXICITY	Down	0.007
KEGG_FC_GAMMA_R_MEDIATED_PHAGOCYTOSIS	Down	0.017
KEGG_INTESTINAL_IMMUNE_NETWORK_FOR_IGA_PRODUCTION	Down	<0.001
KEGG_CHRONIC_MYELOID_LEUKEMIA	Up	0.033

### Genome-wide *microRNA* profiles associated with *MAPKBP1* expression

An analysis of microRNA genome-wide profiles revealed that 78 microRNAs were significantly associated with *MAPKBP1* expression (*P* < 0.05) (supplemental Table S3). *MAPKBP1*^high^ was associated with *miR-155, miR-146a, miR-92a-1, miR-126, miR-133a, miR-25*, and *miR-130a* up-regulation. Up-regulation of *miR-155* was consistent with the gene-expression profiles. *MiR-146a* lost in myelodysplastic syndrome (MDS) with 5q- and related with down-regulation of immune-response pathway [31, 32]. *MiR-92a-1* arouses erythroleukemia through *p53* down-regulation [33]. *MiR-126* promotes survival and inhibits apoptosis of AML cells [34]. *MiR-133a* was up-regulated in CN-AML with *IDH2* codon R172K [35]. *MiR-25* increases somatic cells into induced pluripotent stem cells [36]. *MiR-130a* associated with high expression of *WT1* [32]. Notably *miR-148a* and *miR-193a* were down-regulated. *miR-148a* has recently been shown to target *DNMT3B* [37], the expression of which is an independent unfavourable prognostic factor in older CN-AML patients [18], and it is also associated with *ERG* up-regulation [16]. This was consistent with the gene-expression profiles. We previously found that *miR-193a* targeted *c-kit*, leading to higher expression of this gene, which is also consistent with the observed gene-expression profiles (Figure 4) [38, 39].

### Genome-wide methylation profiling associated with *MAPKBP1* expression

The control of gene expression by DNA methylation has been suggested to play a pivotal role in determining the biological behaviour of cells, and the DNA methylation classifier could predict clinical outcome in AML patients [40, 41]. We therefore assessed whether *MAPKBP1*^high^ and *MAPKBP1*^low^ CN-AML showed different DNA methylation patterns overall, within important cell signalling pathways and individual genes. However, we found no significant differences in DNA methylation with respect to *MAPKBP1* expression in any of these analyses (Supplementary Figure 2, 3, DNA methylation patterns of cell signalling pathways' data not shown).

## DISCUSSION

Our results are of particular interest because a recent paper showed that *MAPKBP1* was an important constitutive activator of *NF-κB* signalling pathway which was required for self-renewal of normal hematopoietic and leukemic stem cells [42, 43]. Leukemogenic fusion genes and gene mutations can induce *NF-κB* cell signalling pathway in AML [44], and small-molecule *NF-κB* pathway inhibitors are cytotoxic for AML blasts [45], and the *SP1/NF-κB* transactivation complex mediated SPARC expression, contributing to leukemogenesis in CN-AML [26]. These findings suggest that expression of *MAPKBP1* may be a prognostic factor in patients with CN-AML.

Our study is the first report on the prognostic relevance of *MAPKBP1* expression in CN-AML, and demonstrates that *MAPKBP1*high is associated with shorter EFS and OS in CN-AML.

*MAPKBP1* was up-regulated in CN-AML compared with normal BM. We found that patients with *MAPKBP1*high were significantly more classified in the M1 FAB subgroups, suggesting that the leukemic cells of the *MAPKBP1*^high^ patients derive from immature cells. We also found that *MAPKBP1*^high^ was associated with the presence of *FLT3-ITD,* higher *ERG, WT1, DNMT3b*, *TCF4* expression, and lower *LEF1* expression, all of which are unfavourable molecular characteristics in CN-AML. Furthermore, the association of *MAPKBP1*^high^ with shorter EFS and OS was confirmed in multivariable analyses adjusting for other known clinical and molecular prognosticators in CN-AML. *MAPKBP1*^high^ was associated with wild type *NPM1* and *FLT3-ITD*, both of which are unfavourable molecular characteristics in CN-AML. These results indicated that *MAPKBP1*^high^ was a surrogate marker for other unfavourably genetic lesions such as the *FLT3-ITD*. Our results suggest that the prognostic impact of *MAPKBP1* expression was most pronounced in the ELN intermediate-I genetic group, and thus *MAPKBP1* expression may be used to further refine risk stratification for these patients.

The mechanisms underlying the association between *MAPKBP1*^high^ and unfavourable treatment outcomes are unclear. In our present study, we analysed gene and microRNA expression, and DNA methylation profiles to identify biological pathways that are associated with *MAPKBP1* expression in CN-AML. Gene sets related to cell proliferation and cell cycle regulation were up-regulated in the CN-AML cells of *MAPKBP1*^high^ patients, and gene sets related to apoptosis were down-regulated. Furthermore, antigen processing and natural killer cell-mediated cytotoxicity, which can lead to immune escape, were down-regulated in CN-AML with *MAPKBP1*high [46, 47]. These changes might contribute to an unfavourable outcome.

The *MAPKBP1*-associated microRNA profile was also noteworthy, as it included nine important *microRNAs* that were differentially expressed in *MAPKBP1*^high^ CN-AML. The up-regulation of *miR-155* was associated with an unfavourable clinical outcome independently in CN-AML, *miR-130a* associated with high expression of *WT1*, and the down-regulation of *miR-148a* and *miR-193a* contributed AML leukemogenesis. However, we found no significant association between *MAPKBP1* expression levels and overall methylation, or the methylation of tumour suppressor genes, or genes involved in important cell signalling pathways.

In summary, our study is the first to provide evidence that *MAPKBP1*^high^ is associated with unfavourable outcomes in CN-AML patients, even after adjusting for most of known molecular risk factors. Because the gene is widely expressed at a high level in CN-AML compared with normal BM, *MAPKBP1* expression can be easily measured. Further qPCR confirmation of microarray expression data could validate these results and made them more reliable. This may therefore be a valuable new marker for risk stratification of CN-AML patients. Moreover, our gene/microRNA expression data from a large cohort of primary CN-AML patients provides insights into the biological changes associated with varying *MAPKBP1* expression levels in CN-AML, and might help direct new therapeutic strategies for CN-AML patients.

## METHODS

### Patients and treatment

One hundred and fifty-seven patients with previously untreated CN-AML (median age, 50 years; range, 16–77 years) were studied, all of whom were received uniform therapeutic treatment based on study protocols of the Dutch-Belgian Hemato-Oncology Cooperative Group (HOVON) between 1990 and 2008 (available at http://www.hovon.nl). The details of therapeutic protocol were shown in supplementary Figure 4 [48]. One hundred thirty patients (83%) were aged <60 years (younger patients) and 27 patients (17%) were ≥ 60 years (older patients). The diagnosis of a normal karyotype was based on conventional cytogenetic examination of at least 20 metaphases from BM. Patients were assessed for *NPM1, CEBPA, N-RAS, K-RAS, IDH1*, and *IDH2* mutations, *FLT3-ITD*, and tyrosine kinase domain mutations (*FLT3-TKD* [D835]). Clinical, cytogenetic and molecular information as well as the gene expression profiles of all primary AML cases could be publicly downloaded at the Gene Expression Omnibus (www.ncbi.nlm.nih.gov/geo, accession number *GSE6891*) [48]. This research was approved by the institutional review boards at Weill Cornell Medical College and Erasmus University Medical Center, and written donor informed consent was obtained in accordance with the Declaration of Helsinki [41]. Another independent validation cohort of 162 CN-AML patients also received uniform therapeutic treatment provided by the multicenter AMLCG-1999 trial was used to validate our findings. These patients received intensive double induction and consolidation chemotherapy. Gene expression data are publicly available (http://www.ncbi.nlm.nih.gov/geo/, accession number *GSE12417*) [49]. The AMLCG-1999 clinical trials were approved by the local institutional review boards, and informed consent from all patients was obtained in accordance with the Declaration of Helsinki [49].

### Microarray analyses

Gene expression and methylation data have been previously published (accession number *GSE1159* [29], *GSE6891* [48] and *GSE12417* [49] for expression, *GSE18700* [41] for methylation). Briefly, gene expression and methylation data were obtained using Affymetrix Human Genome 133 plus 2.0 Gene Chips, Human Genome U133A and HELP methylation arrays [41]. All the design and quality control for microarray experiment were according to the standard Affymetrix protocols. Expression data of microRNA were carried out from The Cancer Genome Atlas (TCGA) obtained by whole-genome high-throughput sequencing, which provided 79 CN-AML patients [50]. Patients with *MAPKBP1* expression values above the median of all patients were classified as having *MAPKBP1*^high^, and the others were considered to have *MAPKBP1*^low^. *ERG, BAALC, LEF1, MN1, EVI1, WT1, DNMT3B*, and *TCF4* expression levels were also determined from the microarray data.

### Statistical analyses

The time from date of diagnosis to removal from study due to absence of complete remission, relapse or death defined EFS, and the time from date of diagnosis to death due to any cause defined OS. Firstly, we subdivided 157 CN-AML patients into four quartiles (Q1: <25%, Q2: 25~50%, Q3: 50~75%, Q4: >75%) based on *MAPKBP1* expression value to determine the best classification method of this group. No significant difference was observed between Q1 and Q2 (Q12, *P* = 0.97), and the same result was also observed between Q3 and Q4 (Q34, *P* = 0.335). However, patients in Q2 and Q3 (Q23, *P* = 0.047) had significant differences (supplementary Figure 5). Secondly, median values of *MAPKBP1* expression were calculated in order to divide patients into high and low expression groups. The Kaplan-Meier method was then used to estimate the association between *MAPKBP1* expression and the EFS and OS, which were further validated using a log-rank test. To investigate the associations between *MAPKBP1* expression levels and clinical, molecular characteristics, the Fisher exact and Wilcoxon rank-sum tests were used in the hypothesis testing for categorical and continuous variables, respectively. In addition, multivariable Cox proportional hazards models were used to study how *MAPKBP1* expression levels associated with EFS and OS in the presence of other known risk factors. According to the two groups divided by *MAPKBP1* expression levels, Student's *t*-test and multiple hypothesis correction (False Discovery Rate, FDR) was used to identify differences in gene-microRNA expression and DNA methylation profiles. The statistical cutoff values were an fold-change (FC) >= 1.5 and an adjusted *P*-value <= 0.05. All analyses were performed using the R 3.1.1 software packages.

## SUPPLEMENTARY MATERIAL, FIGURES AND TABLE



## References

[1] Mrozek K, Marcucci G, Paschka P, Whitman SP, Bloomfield CD (2007). Clinical relevance of mutations and gene-expression changes in adult acute myeloid leukemia with normal cytogenetics: are we ready for a prognostically prioritized molecular classification?. Blood.

[2] Walker A, Marcucci G (2012). Molecular prognostic factors in cytogenetically normal acute myeloid leukemia. Expert review of hematology.

[3] Marcucci G, Mrozek K, Radmacher MD, Garzon R, Bloomfield CD (2011). The prognostic and functional role of microRNAs in acute myeloid leukemia. Blood.

[4] Marcucci G, Yan P, Maharry K, Frankhouser D, Nicolet D, Metzeler KH, Kohlschmidt J, Mrozek K, Wu YZ, Bucci D, Curfman JP, Whitman SP, Eisfeld AK, Mendler JH, Schwind S, Becker H (2014). Epigenetics meets genetics in acute myeloid leukemia: clinical impact of a novel seven-gene score. Journal of clinical oncology: official journal of the American Society of Clinical Oncology.

[5] Becker H, Marcucci G, Maharry K, Radmacher MD, Mrozek K, Margeson D, Whitman SP, Wu YZ, Schwind S, Paschka P, Powell BL, Carter TH, Kolitz JE, Wetzler M, Carroll AJ, Baer MR (2010). Favorable prognostic impact of NPM1 mutations in older patients with cytogenetically normal de novo acute myeloid leukemia and associated gene- and microRNA-expression signatures: a Cancer and Leukemia Group B study. Journal of clinical oncology: official journal of the American Society of Clinical Oncology.

[6] Fasan A, Haferlach C, Alpermann T, Jeromin S, Grossmann V, Eder C, Weissmann S, Dicker F, Kohlmann A, Schindela S, Kern W, Haferlach T, Schnittger S (2014). The role of different genetic subtypes of CEBPA mutated AML. Leukemia.

[7] Marcucci G, Haferlach T, Dohner H (2011). Molecular genetics of adult acute myeloid leukemia: prognostic and therapeutic implications. Journal of clinical oncology: official journal of the American Society of Clinical Oncology.

[8] Paschka P, Marcucci G, Ruppert AS, Whitman SP, Mrozek K, Maharry K, Langer C, Baldus CD, Zhao W, Powell BL, Baer MR, Carroll AJ, Caligiuri MA, Kolitz JE, Larson RA, Bloomfield CD (2008). Wilms' tumor 1 gene mutations independently predict poor outcome in adults with cytogenetically normal acute myeloid leukemia: a cancer and leukemia group B study. Journal of clinical oncology: official journal of the American Society of Clinical Oncology.

[9] Schnittger S, Eder C, Jeromin S, Alpermann T, Fasan A, Grossmann V, Kohlmann A, Illig T, Klopp N, Wichmann HE, Kreuzer KA, Schmid C, Staib P, Peceny R, Schmitz N, Kern W (2013). ASXL1 exon 12 mutations are frequent in AML with intermediate risk karyotype and are independently associated with an adverse outcome. Leukemia.

[10] Dohner K, Tobis K, Ulrich R, Frohling S, Benner A, Schlenk RF, Dohner H (2002). Prognostic significance of partial tandem duplications of the MLL gene in adult patients 16 to 60 years old with acute myeloid leukemia and normal cytogenetics: a study of the Acute Myeloid Leukemia Study Group Ulm. Journal of clinical oncology: official journal of the American Society of Clinical Oncology.

[11] Mendler JH, Maharry K, Radmacher MD, Mrozek K, Becker H, Metzeler KH, Schwind S, Whitman SP, Khalife J, Kohlschmidt J, Nicolet D, Powell BL, Carter TH, Wetzler M, Moore JO, Kolitz JE (2012). RUNX1 mutations are associated with poor outcome in younger and older patients with cytogenetically normal acute myeloid leukemia and with distinct gene and MicroRNA expression signatures. Journal of clinical oncology: official journal of the American Society of Clinical Oncology.

[12] Metzeler KH, Maharry K, Radmacher MD, Mrozek K, Margeson D, Becker H, Curfman J, Holland KB, Schwind S, Whitman SP, Wu YZ, Blum W, Powell BL, Carter TH, Wetzler M, Moore JO (2011). TET2 mutations improve the new European LeukemiaNet risk classification of acute myeloid leukemia: a Cancer and Leukemia Group B study. Journal of clinical oncology: official journal of the American Society of Clinical Oncology.

[13] Thol F, Damm F, Ludeking A, Winschel C, Wagner K, Morgan M, Yun H, Gohring G, Schlegelberger B, Hoelzer D, Lubbert M, Kanz L, Fiedler W, Kirchner H, Heil G, Krauter J (2011). Incidence and prognostic influence of DNMT3A mutations in acute myeloid leukemia. Journal of clinical oncology: official journal of the American Society of Clinical Oncology.

[14] Lyu X, Xin Y, Mi R, Ding J, Wang X, Hu J, Fan R, Wei X, Song Y, Zhao RY (2014). Overexpression of Wilms tumor 1 gene as a negative prognostic indicator in acute myeloid leukemia. PloS one.

[15] Eisfeld AK, Marcucci G, Maharry K, Schwind S, Radmacher MD, Nicolet D, Becker H, Mrozek K, Whitman SP, Metzeler KH, Mendler JH, Wu YZ, Liyanarachchi S, Patel R, Baer MR, Powell BL (2012). miR-3151 interplays with its host gene BAALC and independently affects outcome of patients with cytogenetically normal acute myeloid leukemia. Blood.

[16] Schwind S, Marcucci G, Maharry K, Radmacher MD, Mrozek K, Holland KB, Margeson D, Becker H, Whitman SP, Wu YZ, Metzeler KH, Powell BL, Kolitz JE, Carter TH, Moore JO, Baer MR (2010). BAALC and ERG expression levels are associated with outcome and distinct gene and microRNA expression profiles in older patients with de novo cytogenetically normal acute myeloid leukemia: a Cancer and Leukemia Group B study. Blood.

[17] Schwind S, Marcucci G, Kohlschmidt J, Radmacher MD, Mrozek K, Maharry K, Becker H, Metzeler KH, Whitman SP, Wu YZ, Powell BL, Baer MR, Kolitz JE, Carroll AJ, Larson RA, Caligiuri MA (2011). Low expression of MN1 associates with better treatment response in older patients with de novo cytogenetically normal acute myeloid leukemia. Blood.

[18] Niederwieser C, Kohlschmidt J, Volinia S, Whitman SP, Metzeler KH, Eisfeld AK, Maharry K, Yan P, Frankhouser D, Becker H, Schwind S, Carroll AJ, Nicolet D, Mendler JH, Curfman JP, Wu YZ (2014). Prognostic and biologic significance of DNMT3B expression in older patients with cytogenetically normal primary acute myeloid leukemia. Leukemia.

[19] In ‘t Hout FE, van der Reijden BA, Monteferrario D, Jansen JH, Huls G (2014). High expression of transcription factor 4 (TCF4) is an independent adverse prognostic factor in acute myeloid leukemia that could guide treatment decisions. Haematologica.

[20] Metzeler KH, Heilmeier B, Edmaier KE, Rawat VP, Dufour A, Dohner K, Feuring-Buske M, Braess J, Spiekermann K, Buchner T, Sauerland MC, Dohner H, Hiddemann W, Bohlander SK, Schlenk RF, Bullinger L (2012). High expression of lymphoid enhancer-binding factor-1 (LEF1) is a novel favorable prognostic factor in cytogenetically normal acute myeloid leukemia. Blood.

[21] Marcucci G, Maharry KS, Metzeler KH, Volinia S, Wu YZ, Mrozek K, Nicolet D, Kohlschmidt J, Whitman SP, Mendler JH, Schwind S, Becker H, Eisfeld AK, Carroll AJ, Powell BL, Kolitz JE (2013). Clinical role of microRNAs in cytogenetically normal acute myeloid leukemia: miR-155 upregulation independently identifies high-risk patients. Journal of clinical oncology: official journal of the American Society of Clinical Oncology.

[22] Schwind S, Maharry K, Radmacher MD, Mrozek K, Holland KB, Margeson D, Whitman SP, Hickey C, Becker H, Metzeler KH, Paschka P, Baldus CD, Liu S, Garzon R, Powell BL, Kolitz JE (2010). Prognostic significance of expression of a single microRNA, miR-181a, in cytogenetically normal acute myeloid leukemia: a Cancer and Leukemia Group B study. Journal of clinical oncology: official journal of the American Society of Clinical Oncology.

[23] Lin S, Pan L, Guo S, Wu J, Jin L, Wang JC, Wang S (2013). Prognostic role of microRNA-181a/b in hematological malignancies: a meta-analysis. PloS one.

[24] Breccia M, Alimena G (2010). NF-kappaB as a potential therapeutic target in myelodysplastic syndromes and acute myeloid leukemia. Expert opinion on therapeutic targets.

[25] Karin M (2006). Nuclear factor-kappaB in cancer development and progression. Nature.

[26] Alachkar H, Santhanam R, Maharry K, Metzeler KH, Huang X, Kohlschmidt J, Mendler JH, Benito JM, Hickey C, Neviani P, Dorrance AM, Anghelina M, Khalife J, Tarighat SS, Volinia S, Whitman SP (2014). SPARC promotes leukemic cell growth and predicts acute myeloid leukemia outcome. The Journal of clinical investigation.

[27] Lee SW, Han SI, Kim HH, Lee ZH (2002). TAK1-dependent activation of AP-1 and c-Jun N-terminal kinase by receptor activator of NF-kappaB. Journal of biochemistry and molecular biology.

[28] Yamaguchi T, Miyashita C, Koyano S, Kanda H, Yoshioka K, Shiba T, Takamatsu N, Ito M (2009). JNK-binding protein 1 regulates NF-kappaB activation through TRAF2 and TAK1. Cell biology international.

[29] Valk PJ, Verhaak RG, Beijen MA, Erpelinck CA, Barjesteh van Waalwijk van Doorn-Khosrovani S, Boer JM, Beverloo HB, Moorhouse MJ, van der Spek PJ, Lowenberg B, Delwel R (2004). Prognostically useful gene-expression profiles in acute myeloid leukemia. The New England journal of medicine.

[30] Subramanian A, Tamayo P, Mootha VK, Mukherjee S, Ebert BL, Gillette MA, Paulovich A, Pomeroy SL, Golub TR, Lander ES, Mesirov JP (2005). Gene set enrichment analysis: a knowledge-based approach for interpreting genome-wide expression profiles. Proceedings of the National Academy of Sciences of the United States of America.

[31] Starczynowski DT, Kuchenbauer F, Argiropoulos B, Sung S, Morin R, Muranyi A, Hirst M, Hogge D, Marra M, Wells RA, Buckstein R, Lam W, Humphries RK, Karsan A (2010). Identification of miR-145 and miR-146a as mediators of the 5q- syndrome phenotype. Nature medicine.

[32] Havelange V, Stauffer N, Heaphy CC, Volinia S, Andreeff M, Marcucci G, Croce CM, Garzon R (2011). Functional implications of microRNAs in acute myeloid leukemia by integrating microRNA and messenger RNA expression profiling. Cancer.

[33] Li Y, Vecchiarelli-Federico LM, Li YJ, Egan SE, Spaner D, Hough MR, Ben-David Y (2012). The miR-17-92 cluster expands multipotent hematopoietic progenitors whereas imbalanced expression of its individual oncogenic miRNAs promotes leukemia in mice. Blood.

[34] Li Z, Lu J, Sun M, Mi S, Zhang H, Luo RT, Chen P, Wang Y, Yan M, Qian Z, Neilly MB, Jin J, Zhang Y, Bohlander SK, Zhang DE, Larson RA (2008). Distinct microRNA expression profiles in acute myeloid leukemia with common translocations. Proceedings of the National Academy of Sciences of the United States of America.

[35] Marcucci G, Maharry K, Wu YZ, Radmacher MD, Mrozek K, Margeson D, Holland KB, Whitman SP, Becker H, Schwind S, Metzeler KH, Powell BL, Carter TH, Kolitz JE, Wetzler M, Carroll AJ (2010). IDH1 and IDH2 gene mutations identify novel molecular subsets within de novo cytogenetically normal acute myeloid leukemia: a Cancer and Leukemia Group B study. Journal of clinical oncology: official journal of the American Society of Clinical Oncology.

[36] Lu D, Davis MP, Abreu-Goodger C, Wang W, Campos LS, Siede J, Vigorito E, Skarnes WC, Dunham I, Enright AJ, Liu P (2012). MiR-25 regulates Wwp2 and Fbxw7 and promotes reprogramming of mouse fibroblast cells to iPSCs. PloS one.

[37] Duursma AM, Kedde M, Schrier M, le Sage C, Agami R (2008). miR-148 targets human DNMT3b protein coding region. RNA.

[38] Li Y, Gao L, Luo X, Wang L, Gao X, Wang W, Sun J, Dou L, Li J, Xu C, Zhou M, Jiang M, Zhou J, Caligiuri MA, Nervi C, Bloomfield CD (2013). Epigenetic silencing of microRNA-193a contributes to leukemogenesis in t(8;21) acute myeloid leukemia by activating the PTEN/PI3K signal pathway. Blood.

[39] Gao XN, Lin J, Li YH, Gao L, Wang XR, Wang W, Kang HY, Yan GT, Wang LL, Yu L (2011). MicroRNA-193a represses c-kit expression and functions as a methylation-silenced tumor suppressor in acute myeloid leukemia. Oncogene.

[40] Schoofs T, Berdel WE, Muller-Tidow C (2014). Origins of aberrant DNA methylation in acute myeloid leukemia. Leukemia.

[41] Figueroa ME, Lugthart S, Li Y, Erpelinck-Verschueren C, Deng X, Christos PJ, Schifano E, Booth J, van Putten W, Skrabanek L, Campagne F, Mazumdar M, Greally JM, Valk PJ, Lowenberg B, Delwel R (2010). DNA methylation signatures identify biologically distinct subtypes in acute myeloid leukemia. Cancer cell.

[42] Zhao C, Xiu Y, Ashton J, Xing L, Morita Y, Jordan CT, Boyce BF (2012). Noncanonical NF-kappaB signaling regulates hematopoietic stem cell self-renewal and microenvironment interactions. Stem Cells.

[43] Guzman ML, Neering SJ, Upchurch D, Grimes B, Howard DS, Rizzieri DA, Luger SM, Jordan CT (2001). Nuclear factor-kappaB is constitutively activated in primitive human acute myelogenous leukemia cells. Blood.

[44] Goyama S, Mulloy JC (2013). NF-kappaB: a coordinator for epigenetic regulation by MLL. Cancer cell.

[45] Herman SE, Mustafa RZ, Gyamfi JA, Pittaluga S, Chang S, Chang B, Farooqui M, Wiestner A (2014). Ibrutinib inhibits BCR and NF-kappaB signaling and reduces tumor proliferation in tissue-resident cells of patients with CLL. Blood.

[46] Elias S, Yamin R, Golomb L, Tsukerman P, Stanietsky-Kaynan N, Ben-Yehuda D, Mandelboim O (2014). Immune evasion by oncogenic proteins of acute myeloid leukemia. Blood.

[47] Lion E, Willemen Y, Berneman ZN, Van Tendeloo VF, Smits EL (2012). Natural killer cell immune escape in acute myeloid leukemia. Leukemia.

[48] Verhaak RG, Wouters BJ, Erpelinck CA, Abbas S, Beverloo HB, Lugthart S, Lowenberg B, Delwel R, Valk PJ (2009). Prediction of molecular subtypes in acute myeloid leukemia based on gene expression profiling. Haematologica.

[49] Metzeler KH, Hummel M, Bloomfield CD, Spiekermann K, Braess J, Sauerland MC, Heinecke A, Radmacher M, Marcucci G, Whitman SP, Maharry K, Paschka P, Larson RA, Berdel WE, Buchner T, Wormann B (2008). An 86-probe-set gene-expression signature predicts survival in cytogenetically normal acute myeloid leukemia. Blood.

[50] (2013). >Genomic epigenomic landscapes of adult de novo acute myeloid leukemia. The New England journal of medicine.

