# Supplementary material for: Mitogen-activated protein kinase binding protein 1 (MAPKBP1) is an unfavorable prognostic biomarker in cytogenetically normal acute myeloid leukemia
Source: Oncotarget. 2015 Mar 10;6(10):8144–54. doi: 10.18632/oncotarget.3519 (PMC4480741; doi:10.18632/oncotarget.3519)
Supplement: Supplementary file 1 [file oncotarget-06-8144-s001.pdf]

Mitogen-activated protein kinase binding protein 1 (*MAPKBP1*) is an unfavorable prognostic biomarker in cytogenetically normal acute myeloid leukemia

Supplementary Material

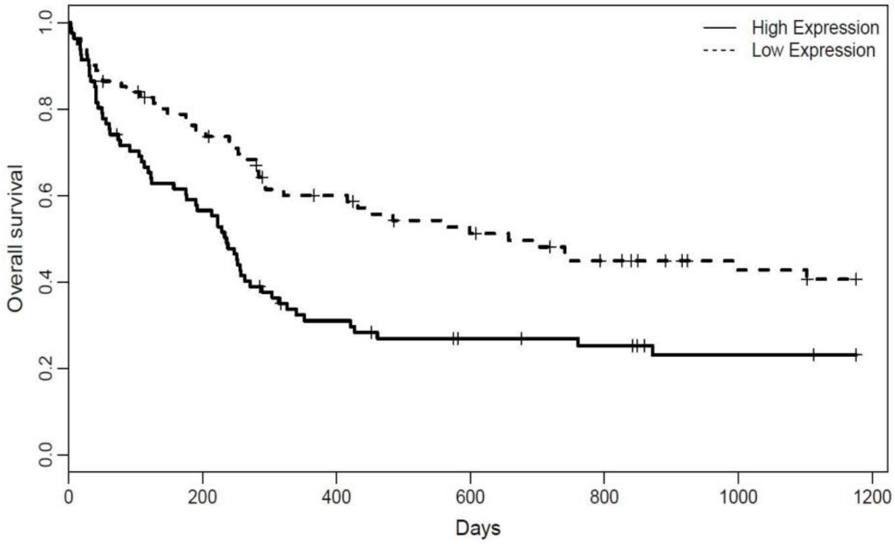

Figure S1. OS of CN-AML patients in the validating cohort.

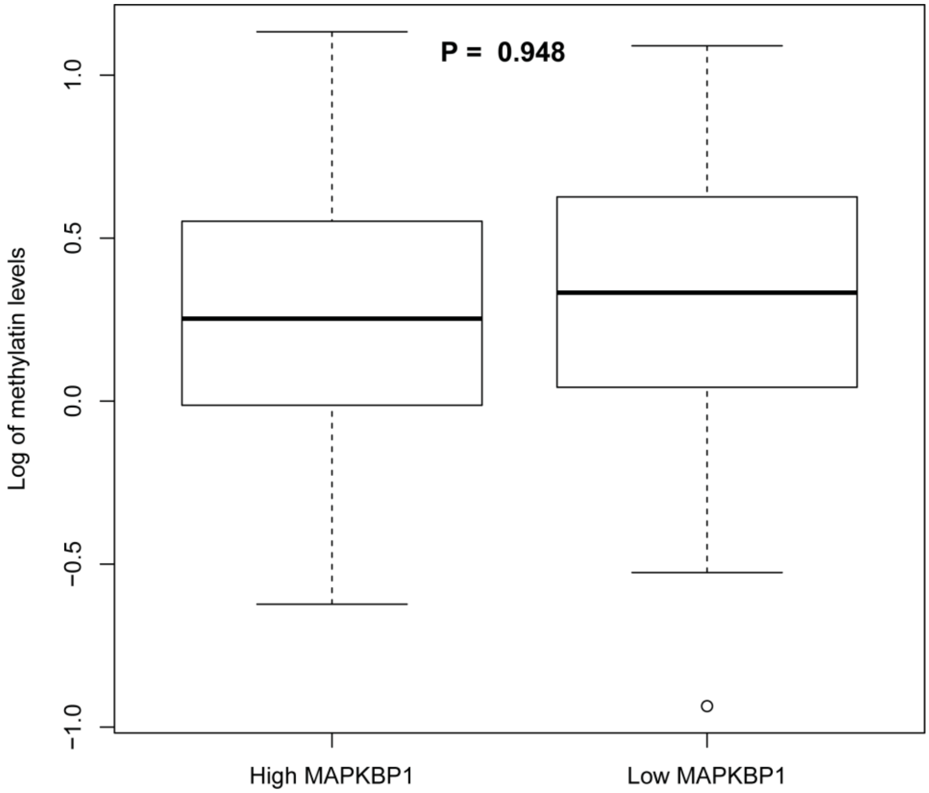

Figure S2. Genome-wide DNA methylation levels between *MAPKBP1*^{high} and *MAPKBP1*^{low} patients.

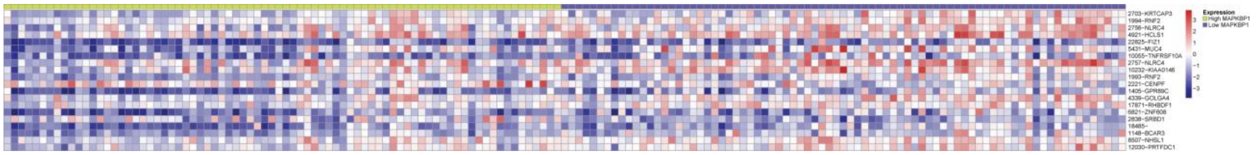

Figure S3. Heatmap of individual genes' promoter methylation levels difference between *MAPKBP1*$^{\mathbf{high}}$ and *MAPKBP1*$^{\mathbf{low}}$ patients.

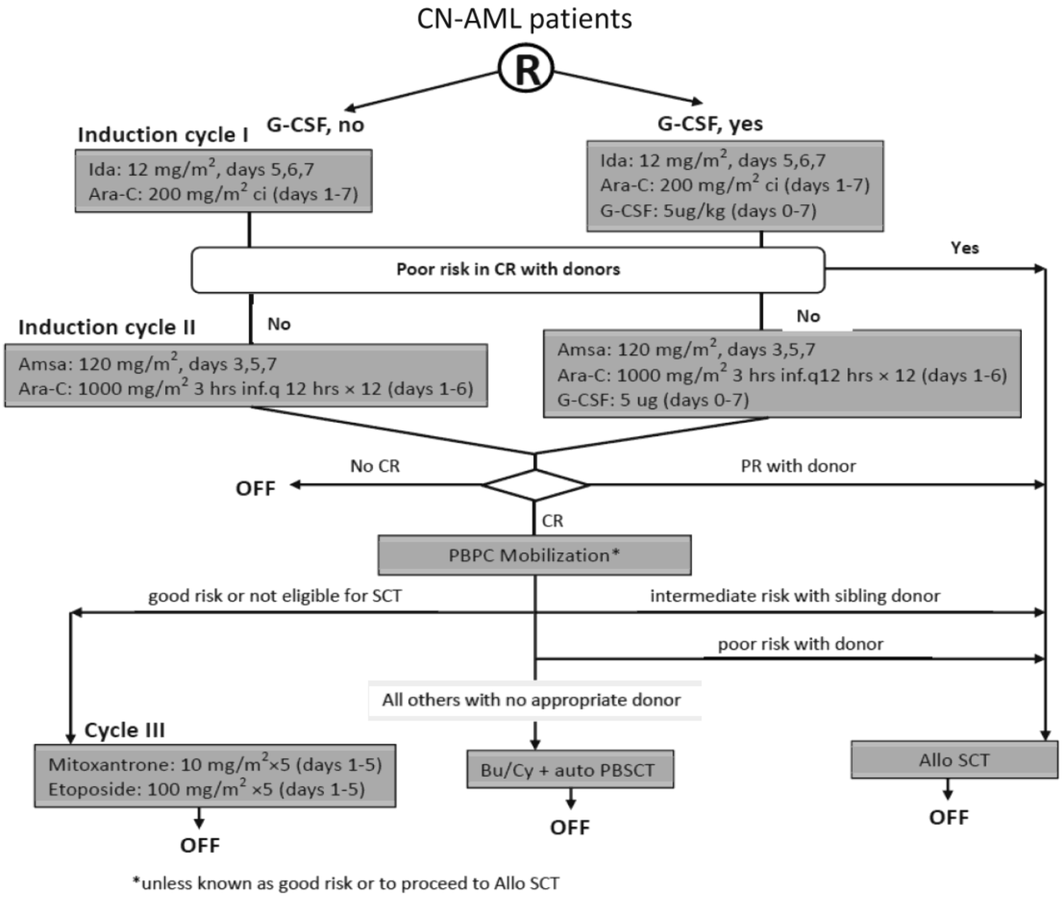

Figure S4. Therapeutic strategies of CN-AML patients.

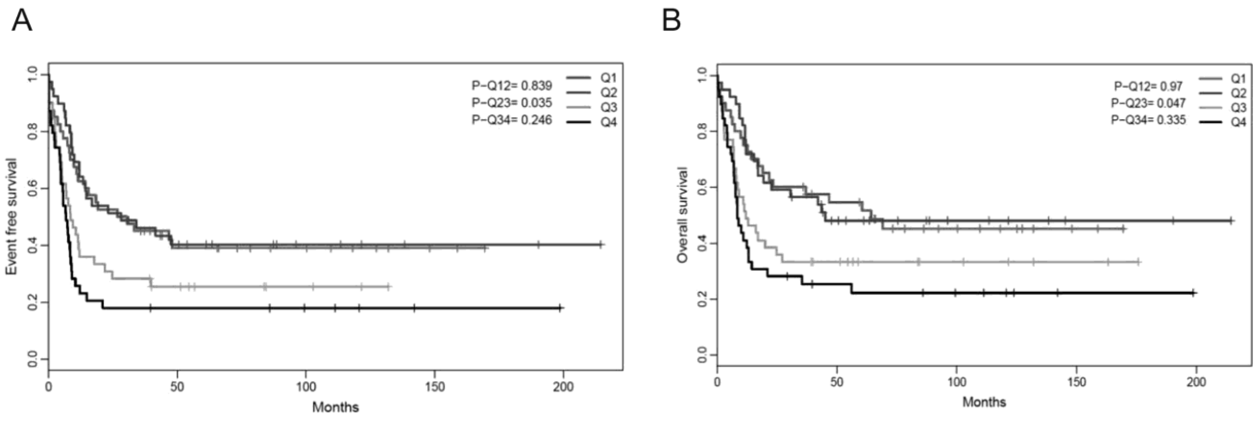

Figure S5. Associations of *MAPKBP1* expression with clinical outcome according to four quartiles. (A) EFS and (B) OS in the entire cohort of 157 CN-AML cases.

Table S1. Patients' characteristics in the validating cohort of CN-AML according to the *MAPKBP1* expression

Variable	$MAPKBP1^{high}$, n=81	$MAPKBP1^{low}$,n=81	P
Median age. y (range)	60 (17-81)	55 (19-83)	0.542
Median OS. d (range)	233 (1-1176)	425 (1-1176)	0.004
FAB subtype, no.			
M0	3	2	1
M1	32	13	0.001
M2	20	25	0.483
M3	0	0	
M4	20	22	0.858
M5	6	13	0.14
M6	0	6	0.0284
High ERG, no.	57	24	P<0.001
High BAALC, no.	44	37	0.346
High LEF1, no.	25	56	P<0.001
High MN1, no.	48	33	0.028
High WT1, no.	55	29	P<0.001
High DNMT3B, no.	56	25	P<0.001
High TCF4, no.	56	25	P<0.001

CN-AML indicates cytogenetically normal acute myeloid leukemia; FAB, French-American-British classification.

High *ERG*，*BAALC*，*LEF1, MN1, WT1*, *DNMT3B* and *TCF4* expression were defined as an expression level above the median of all samples, respectively.
